# Reproductive Number and Serial Interval of the First Wave of Influenza A(H1N1)pdm09 Virus in South Africa

**DOI:** 10.1371/journal.pone.0049482

**Published:** 2012-11-16

**Authors:** Brett N. Archer, Stefano Tempia, Laura F. White, Marcello Pagano, Cheryl Cohen

**Affiliations:** 1 National Institute for Communicable Diseases (NICD), National Health Laboratory Service (NHLS), Johannesburg, Gauteng, South Africa; 2 United States Centers for Disease Control and Prevention, Attaché to the National Institute for Communicable Diseases (NICD), National Health Laboratory Service (NHLS), Johannesburg, Gauteng, South Africa; 3 Department of Biostatistics, School of Public Health, Boston University, Boston, Massachusetts, United States of America; 4 School of Public Health, Harvard University, Cambridge, Massachusetts, United States of America; 5 School of Public Health, University of Witwatersrand, Johannesburg, Gauteng, South Africa; University of Hong Kong, Hong Kong

## Abstract

**Background/Objective:**

Describing transmissibility parameters of past pandemics from diverse geographic sites remains critical to planning responses to future outbreaks. We characterize the transmissibility of influenza A(H1N1)pdm09 (hereafter pH1N1) in South Africa during 2009 by estimating the serial interval (SI), the initial effective reproductive number (initial *R_t_*) and the temporal variation of *R_t_*.

**Methods:**

We make use of data from a central registry of all pH1N1 laboratory-confirmed cases detected throughout South Africa. Whenever date of symptom onset is missing, we estimate it from the date of specimen collection using a multiple imputation approach repeated 100 times for each missing value. We apply a likelihood-based method (method 1) for simultaneous estimation of initial *R_t_* and the SI; estimate initial *R_t_* from SI distributions established from prior field studies (method 2); and the Wallinga and Teunis method (method 3) to model the temporal variation of *R_t_*.

**Results:**

12,360 confirmed pH1N1 cases were reported in the central registry. During the period of exponential growth of the epidemic (June 21 to August 3, 2009), we simultaneously estimate a mean *R_t_* of 1.47 (95% CI: 1.30–1.72) and mean SI of 2.78 days (95% CI: 1.80–3.75) (method 1). Field studies found a mean SI of 2.3 days between primary cases and laboratory-confirmed secondary cases, and 2.7 days when considering both suspected and confirmed secondary cases. Incorporating the SI estimate from field studies using laboratory-confirmed cases, we found an initial *R_t_* of 1.43 (95% CI: 1.38–1.49) (method 2). The mean *R_t_* peaked at 2.91 (95% CI: 0.85–2.91) on June 21, as the epidemic commenced, and *R_t_*>1 was sustained until August 22 (method 3).

**Conclusions:**

Transmissibility characteristics of pH1N1 in South Africa are similar to estimates reported by countries outside of Africa. Estimations using the likelihood-based method are in agreement with field findings.

## Introduction

During 2009, the emergence and worldwide spread of influenza A(H1N1)pdm09 (pH1N1) was observed [1]. While a rapid and timely estimation of the transmission parameters of this novel virus played an important role in informing transmission potential and mitigation interventions during the 2009 pandemic period, the post-pandemic documentation of these parameters is equally important as many previous estimates were established from analyses conducted during the early stages of epidemics and often from preliminary data [2], [3]. Additionally enhancing our knowledge of past pandemics assists in providing greater insight to prepare and respond in future outbreaks.

Four key measures are typically used to describe the transmissibility of an infectious disease. First, the serial interval (SI) describes the mean time between illness onset of two successive cases in the chain of transmission. Second, the secondary attack rate (SAR) describes the proportion of susceptible contacts that acquire infection from an infectious person. Third, the basic reproductive number (*R_0_*) is defined as the average number of secondary cases per primary case in an idealised entirely susceptible population in the absence of control measures. Finally, the effective reproductive number (*R_t_*) at any given time point represents the actual average number of secondary cases per primary case observed in a population. *R_t_* reflects the impact of control measures and the depletion of susceptible persons over time. The initial *R_t_* may approximate *R_0_* in pandemic situations. [2]–[5].

Previously published estimates of pH1N1 transmission parameters vary by study setting and methods employed. The majority of studies found the mean SI of pH1N1 to range from 2.5–3.3 days [2], [6]–[11]; however, Canada and Texas reported a longer SI of 4–5 days, respectively [12], [13]. Estimates of the *R*_0_ of pandemic influenza from the USA range from 1.3–2.3 [2], [9], [11]. Estimates from Mexico range from 1.4–2.9 [2], [14], [15]. Outside of North America, *R_0_* estimates include: Australia (mean 2.4) [16], Canada (mean 2.62) [12], Thailand (mean 2.07) [17], Peru (range 1.2–1.7) [18] and New Zealand (mean 1.96) [19]. Finally, Japan revised their mean *R_0_* estimates from 2.3 to 1.28 after repeating analyses later in the pandemic [20]; thus demonstrating a need to revisit revised and more complete datasets. A variation in *R_t_* with progression of the pandemic was observed in Mexico, averaging at 1.47 (based on a negative binomial model) [14], but peaking between 2.1–4.0 depending on the generation interval chosen [21].

In a previous work, we estimated the SAR and SI of pH1N1 among the first 100 cases detected in South Africa by prospectively examining virus transmission between household contacts [22]. We found a SAR of 10% and a mean SI of 2.3 days (SD ±1.3, range 1–5) between successive laboratory-confirmed cases in the transmission chain. When additionally including suspected secondary cases into the analysis, the SAR increased to 17% and the SI to 2.7 days (SD ±1.5, range 1–6). In this work we incorporate data collected on all laboratory-confirmed cases detected during the 2009 pH1N1 epidemic in South Africa with the aim of describing the transmissibility characteristics (initial *R_t_* and temporal variation of *R_t_*) of the epidemic in the country and compare its dynamics with those observed in other countries in the same year.

## Methods

### Data

During 2009, the National Institute for Communicable Diseases (NICD), of the National Health Laboratory Service (NHLS), South Africa, maintained a central registry of all pH1N1 laboratory-confirmed cases detected throughout the country. The methodology of collating this data has previously been described in detail [23]. Briefly, we collated individual case-based data from all laboratories offering pH1N1 testing throughout South Africa, which included patient age, sex, dates of illness onset and specimen collection, and the administrative location (province) of the healthcare facility where the patient presented. Testing was performed by accredited laboratories, including: the National Influenza Centre (NICD-NHLS), NHLS public-sector laboratories or private-sector laboratories. All testing laboratories performed detection and characterisation of pH1N1 virus by real-time PCR by either the protocol developed by the WHO Collaborating Centre for Influenza, U.S. Centers for Disease Control and Prevention [24], or using commercially available kits.

### Imputation of Missing Data

Wherever the date of symptom onset was missing, we estimated it from the date of specimen collection using a multiple imputation approach. Firstly, we modelled the lag time from date of symptoms onset to date of specimens collection from cases with complete data via a Poisson regression model using predictors significant at p<0.05. The covariates assessed in the model were patient age, gender, province, date of specimen collection, and collection of a specimen on a weekend day (i.e. Saturday or Sunday). Secondly we obtained an estimated lag-time for each observation with missing date of symptoms onset using a random sampling process from a Poisson distribution centred on the predicted value from the Poisson regression model. A Poisson distribution was selected to model count data. Thirdly we imputed missing dates of symptoms onset by subtracting the estimated lag-time from the date of specimen collection. The imputation process was repeated 100 times for each missing value, creating 100 datasets with information on the onset date (imputed or observed) for 12,630 laboratory-confirmed cases.

### Estimation of Intial *R_t_* and Temporal Variation in *R_t_*

We based the estimation of initial *R_t_* and temporal variation of *R_t_* on date of symptoms onset (observed and imputed). In all analyses we modelled the SI via a multinomial distribution. When estimating initial *R_t_*, we focus our analysis on the exponential growth phase of the epidemic in South Africa (i.e. the period from the first occurrence of five consecutive days with confirmed cases reported to the epidemic peak). The parameters were estimated using three methods:

#### Method 1

We make use of the likelihood-based method for the simultaneous estimation of initial *R_t_* and the SI described by White and Pagano (2008) [25]. This method is well suited for estimation of initial *R_t_* and SI in real-time with observed aggregated daily counts of new cases, denoted by *N = (N_0_, N_1_…,N_T_)* where *T* is the last day of observation and *N_0_* are the initial number of seed cases that begin the outbreak. The *N_i_* are assumed to be composed of a mixture of cases that were generated by the previous *k* days, where *k* is the maximal value of the serial interval. We denote these as *X_j_*, the number of cases that appear on day *i* that were infected by individuals with onset of symptoms on day *j*. We assume that the number of infectees generated by infectors with symptoms on day *j* follows a Poisson distribution with parameter *R_t_N_j_*. Additionally, *X_j_* = *(X_j,j+1_*, *X_j,j+2_*…,*X_j,j+k+1_)*, the vector of cases infected by the *N_j_* individuals, follows a multinomial distribution with parameters *p*, *k* and *X_j_*. Here *p* is a vector of probabilities that denotes the serial interval distribution. Using these assumptions, the following likelihood is obtained:
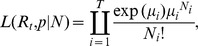
where 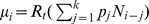.

Parameter estimates are obtained using maximum likelihood methods. For this method we used 6 days as the maximal value of the SI (*k*), which is consistent with the length of the SI observed in field investigations in South Africa [22]. In addition we implemented a sensitivity analysis to assess the variation of the initial *R_t_* estimates vis-à-vis *k* values of 4 days and 8 days, respectively.

**Figure 1:**
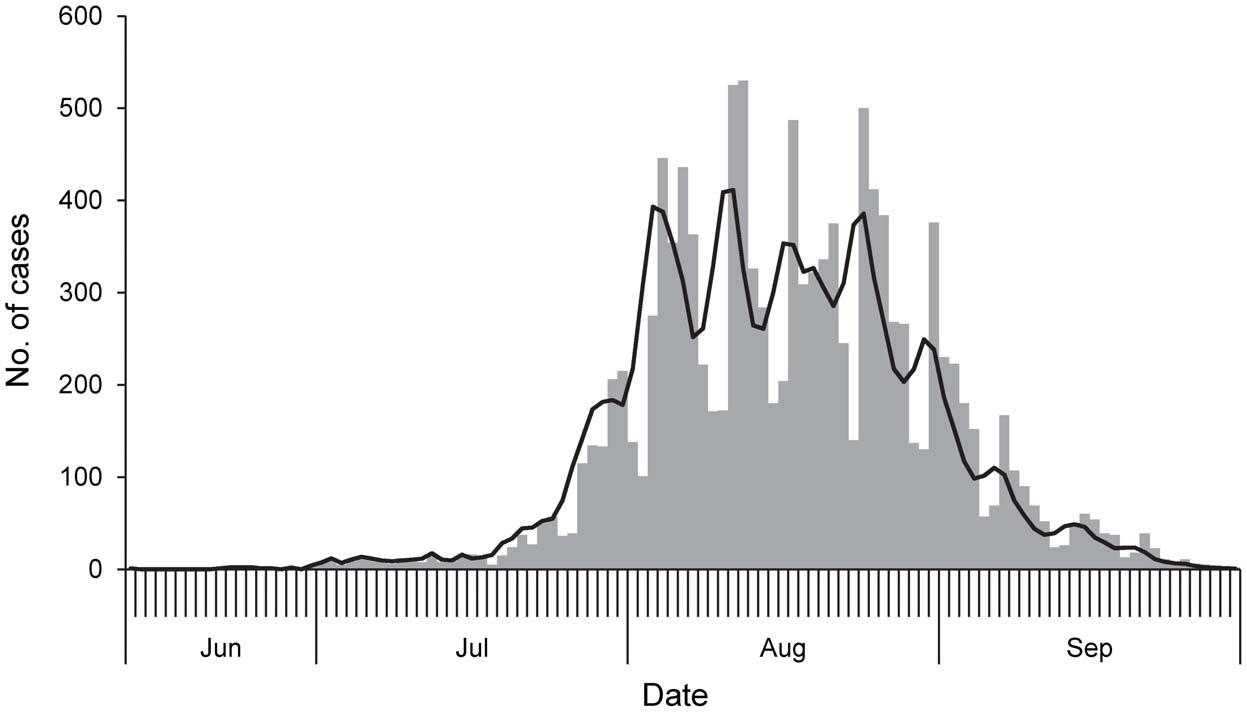
Epidemic curve of laboratory-confirmed influenza A(H1N1)pdm09 cases, South Africa, June 12 to September 30, 2009. Bars show original recorded data applying date of symptom onset where available (n = 758) and substitute by date of specimen collection where onset was unavailable (total n = 12,526). The line shows imputed data where date of symptom onset for missing case-based data was obtained by multiple imputations adjusted by provincial location of specimen collection and the occurrence of a case on a weekend day (n = 12,491).

**Table 1:** Observed lag-time between date of symptom onset and date of specimen collection, incidence rate ratio (IRR) and significance value of the covariates significant in the Poisson regression model.

Factor	Observed Lag-Time Mean (Std. dev.)	Model IRR	p-value
**Province**			<0.001^a^
Eastern Cape	2.1 (2.0)	–	–
Free State	1.9 (1.7)	0.90	0.620
Gauteng	1.7 (2.0)	0.78	0.140
KwaZulu-Natal	3.7 (3.4)	1.73	0.003
Limpopo	1.4 (1.0)	0.66	0.047
Mpumalanga	1.3 (1.5)	0.59	0.042
Northern Cape	2.1 (1.9)	0.95	0.797
North West	1.6 (1.1)	0.80	0.575
Western Cape	1.0 (1.9)	0.50	<0.001
**Day of specimen collection**			
Week day	1.7 (2.1)	–	–
Weekend day	1.1 (1.9)	0.75	0.003

a Pooled p-value for province covariate.

#### Method 2

We assume a known distribution of the SI in South Africa and we estimate the initial *R_t_* using the maximum likelihood estimator for known SI described by White and Pagano (2008) [9], [25]. The estimator of initial *R_t_* in this case is a modification of Method 1 and is given by:
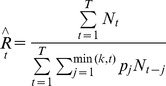



For this analysis we use the two SI distributions observed from investigations of the first 100 pH1N1 cases in South Africa [22]: (1) the SI distribution between primary cases and laboratory-confirmed secondary cases only (39%, 24%, 14%, 17%, 3% and 3% for day 1 to 6 respectively), and (2) the SI distribution between primary cases and suspected plus laboratory-confirmed secondary cases (30%, 17%, 20%, 23%, 7% and 3% for day 1 to 6 respectively). We consider suspected secondary cases, individuals that developed ILI symptoms within 14 days from the symptom onset of a confirmed index case within the same household.

#### Method 3

We make use of the Wallinga and Teunis’ method for estimation of *R_t_* from the imputed data [26]. This method uses the daily case counts of cases and assumes the serial interval is known. We make the same assumptions for the serial interval as in method 2. The method calculates the relative probability a case on day *i* infects a case on day *j* as:
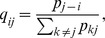
where *p_k_* is the probability of a serial interval of length *k*.

Then the estimate for the reproductive number for case *i*, is:
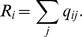



This method requires that we make use of the entire epidemic curve. We calculate *R_t_* as the average of the *R_i_* when *i* is in the epidemic period, as previously defined.

Estimates are reported as the means across the 100 imputations. For all estimates, we calculate bootstrap confidence intervals as has been described previously [9], [26]. We combine the results from all 100 imputations to obtain a confidence interval that incorporates both imputation error, as well as random error [27].

All analyses were performed using R version 2.14.

**Figure 2:**
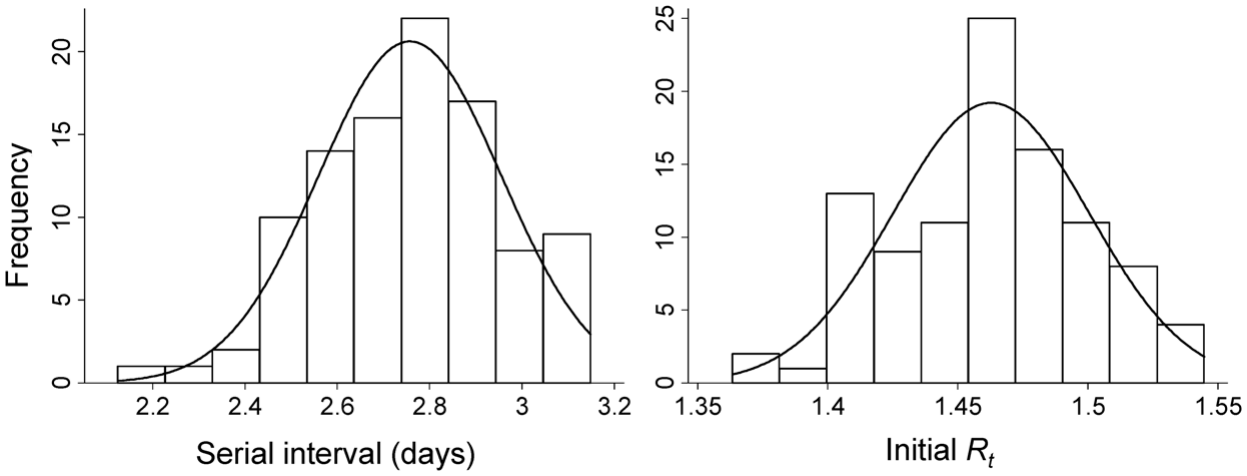
Distribution of serial interval and initial effective reproductive number (*R_t_*) across 100 simulations for the influenza A(H1N1)pdm09 epidemic in South Africa using the likelihood-based simultaneous estimation method (method 1).

**Figure 3:**
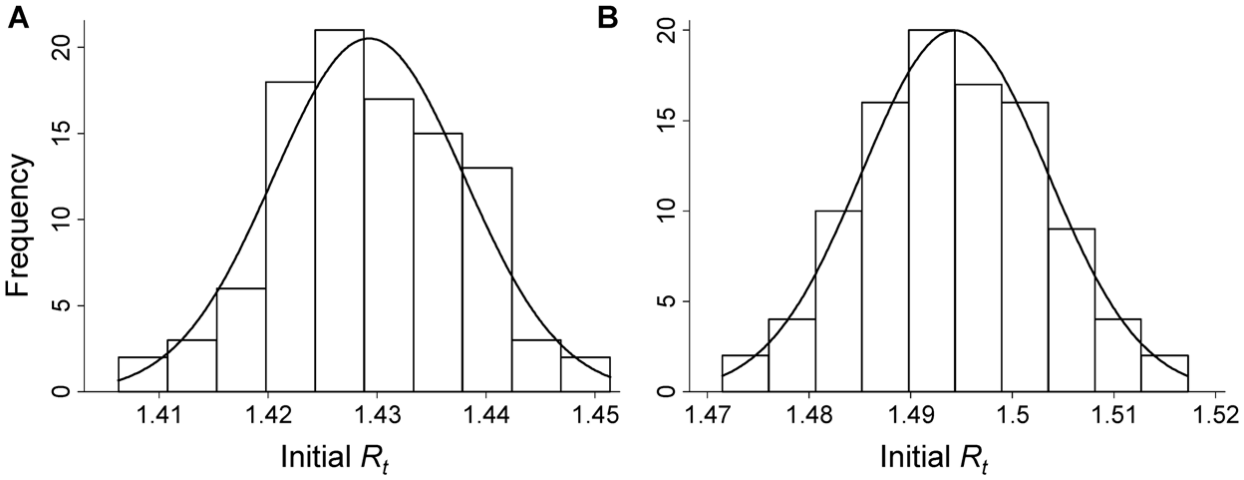
Distribution of the initial effective reproduction number (*R_t_*) across 100 simulations for the pandemic influenza A(H1N1)pdm09 epidemic in South Africa, assuming known serial interval (SI) estimates derived from (A) confirmed secondary cases only (SI: 2.3 days) and (B) confirmed plus suspected secondary cases (SI: 2.7 days) in the transmission chain (method 2).

## Results

### Data and Imputation

12,630 laboratory-confirmed pH1N1 cases were captured by the South African central registry during 2009. The overall demographic, spatial and temporal distribution of these cases has been previously described [23]. Data on date of symptom onset was available for 758 (6%) cases and date of specimen collection for 12,500 (99%) cases. The first case reported illness onset of June 12, 2009 and the epidemic peaked on week 32 (August 3–9, 2009) (Figure 1). The epidemic growth period (when sustained transmission began) started on June 21 (range in imputations: June 20–21) and ended on August 11 (range: August 4–25).

**Figure 4:**
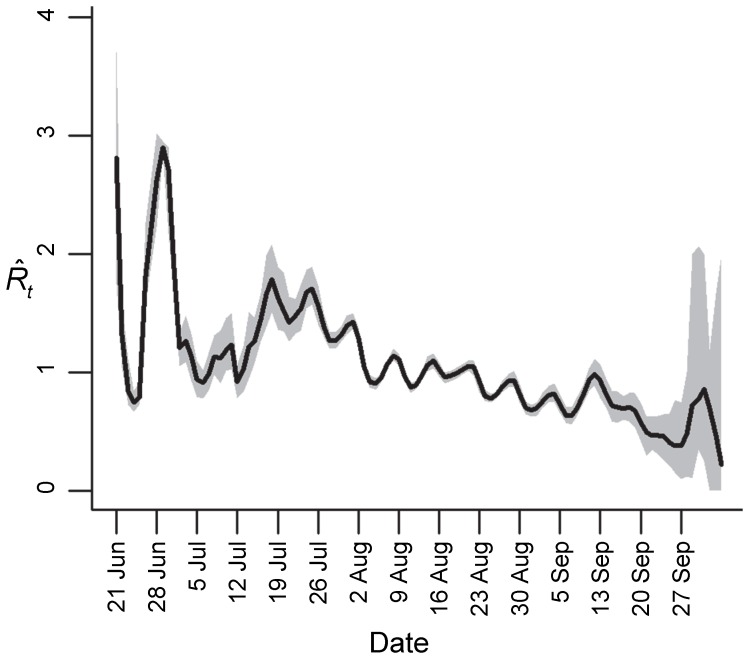
Temporal variation in the mean effective reproductive number (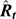) of influenza A(H1N1)pdm09 in South Africa, June 15 to October 4, 2009 (method 3).

The lag-time between symptom onset and specimen collection was significantly associated with the provincial location of specimen collection, as well as the collection of a specimen on a weekend day (Table 1). We used these two covariates in the multiple-imputation to predict the date of symptom onset where missing for all cases (Figure 1). Other available variables, including date of specimen collection (period during the epidemic), patient age and sex were not significantly associated with the lag-time between symptom onset and specimen collection and, therefore, not included in the final model. Analyses to simultaneously estimate initial *R_t_* and serial interval, and estimate initial *R_t_* given a known serial interval, were performed over the exponential growth phase of the epidemic from June 21 to August 3, 2009.

### Simultaneous Estimation of *R_t_* and Serial Interval

Using the likelihood-based method to simultaneously estimate initial *R_t_* and the SI across 100 imputations of the dataset (Method 1), we estimated a 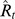 of 1.47 (95% CI: 1.30–1.72) and a mean SI of 2.78 days (95% CI: 1.80–3.75) (Figure 2). 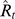 estimates ranged from 1.31 (95% CI: 1.21–1.48) to 1.54 (95% CI: 1.37–2.03) when the maximal value of the SI ranged from 4 to 8 days.

### Estimation of *R_t_* Assuming Known Serial Intervals

We first utilised the SI established from the aforementioned field investigations of the initial 100 cases in estimating *R_t_*, as described in method 2. When performing the analysis using the SI distribution observed for laboratory-confirmed pH1N1 secondary cases only (mean 2.3 days, SD ±1.3, range 1–5) [22], we found an initial 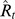 of 1.43 (95% CI: 1.38–1.49) (Figure 3A). When performing the analysis using the SI distribution observed for both confirmed and suspected secondary cases (mean 2.7 days, SD ±1.5, range 1–6) [22], we found an initial 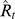 of 1.49 (95% CI: 1.44–1.55) (Figure 3B).

### Estimation of *R_t_*

Figure 4 shows the variation in 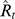 with the progression of the outbreak over time. We observed relatively high 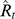 values following the introduction of pH1N1 virus into South Africa, corresponding to high rates of transmission and exponential growth of the local epidemic during this period. 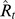 peaked on the first day of the epidemic growth period (June 21) at 2.91 (95% CI: 0.85–3.99). 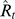 began to drop from July 27 onward and remained consistently below one after August 22. This corresponds with the decline in the daily incidence of new cases detected. Averaging the *R_t_* values obtained during the epidemic growth period (June 21 to August 3, 2009), we estimate initial *R_t_* to be 1.42 (95% CI: 1.20–1.71).

## Discussion

Utilising temporal data on illness onset and specimen collection, and the epidemic curve derived from these data, we provide estimates of the transmissibility parameters of pH1N1 during the first wave experienced in South Africa. Our results focus primarily on the use of analytical techniques to estimate initial *R_t_* and SI without incorporating contact tracing or household transmission studies. However, when parameters from field studies are available, we show that these can be incorporated to provide robust estimates of transmission parameters. We found that initial *R_t_* estimates established using the likelihood-based method for the simultaneous estimation of *R_t_* and SI (method 1: initial 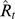: 1.47, SI: 2.78 days) are in agreement with those obtained using SI observed in field investigations [22] (method 2: initial 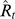: 1.43 and 1.49 using observed SI for laboratory confirmed or laboratory confirmed and suspected cases respectively). In addition, the mean SI estimate obtained with method 1 (2.78 days) is in agreement with field findings (SI: 2.3–2.7 days using observed SI for laboratory confirmed or laboratory confirmed and suspected cases respectively). Previous estimates of initial *R_t_* and the mean SI for pH1N1 have ranged between 1.3–2.9 and 2.5–3.3 days, respectively [2], [6]–[11], [14]–[19]. Our estimates are consistent with these findings, regardless of the method used for the analysis and despite difference in climate, demography and health systems across these countries. It appears that once established, the transmission characteristics of pH1N1 are very consistent. Differences in transmission rates may occur within smaller subgroups of the overall population; however, this has not been well-studied.

Previous estimates of the epidemiological parameters of seasonal influenza epidemics found a SI = 2–4 days [28]–[30], and a *R_t_* a little over 1 with slight variation between climates; *R_t_* = 1.03 in Brazil [31] versus *R_t_* = 1.1–1.3 in more temperate climates [32]. A number of studies have retrospectively estimated the transmissibility of influenza pandemics. During the 1918 Spanish influenza A(H1N1) pandemic, when assuming a SI = 4 days, *R_0_* estimates range from 2.0–4.3 in community settings [33], [34], and even higher values (*R_0_* = 2.6–10.6) in confined settings such as ships and prisons [34]. A separate analysis predicted a slightly lower SI of 3.3 in community settings and a SI of 3.81 in confined settings during the 1918 pandemic, and subsequently estimated *R_0_* values of 1.34–3.21 and 4.97 in these respective settings [35]. *R_0_* estimates from the 1957 Asian influenza A(H2N2) pandemic range from 1.65–1.68 [36], [37]. During the first wave of the 1968–1969 Hong Kong influenza A(H3N2) pandemic, estimates of *R_0_* range from 1.06–2.06 and increased to 1.21–3.58 during the second wave [38].

Given our findings, the overall transmissibility of pH1N1 in South African during 2009 was more similar to that of seasonal influenza strains than the 1918 pandemic, and comparable to lower end estimates of the latter pandemics. However, by showing variation in transmissibility with time, we demonstrate that shortly after introduction of pH1N1 into the country, transmission of the virus reached an 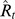 of 2.9, resulting in exponential growth of the local epidemic and widespread illness. Nonetheless, we show that after a period of less than 2 months of heightened transmission, 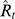 dropped below 1, corresponding to a decline in the incidence of new cases; likely a result of a combination of herd immunity, public health infection control measures and climate impact on virus transmission.

There are several limitations in this analysis which merit discussion. First, we assume that all cases are known and reported. It has been shown previously that, if cases are not reported, this may bias estimates generated using this method [39]. If the proportion of cases reported remains consistent over the study, then the estimates of transmissibility will not be biased; however, if the reporting fraction varies through time, then biased estimates of the reproductive number and serial interval may result. Likewise, variation in case ascertainment with time may bias our estimates of the temporal variation of *R_t_*. Generally higher reporting rates may be anticipated in the early phase, with reporting fatigue later becoming a factor. Secondly, data for this study are derived from laboratory-based surveillance data from several regions across South Africa; a large and diverse country. Our findings do not incorporate heterogeneities (such as spatial and demographic differences) that likely exist in transmission patterns, or assess the degree to which these impact aggregate measures of initial *R_t_*. Methodologies that incorporate heterogeneities inherent in public health data warrant further study.

Despite these limitations, the post-pandemic estimates presented here add to the body of knowledge of pH1N1 transmissibility parameters, which were previously dominated by estimates from developed nations and often based on preliminary data. It remains important that revised parameters, from complete datasets and diverse geographies, are incorporated into planning mitigation strategies for future pandemics. Nonetheless, the methods used in this study would be adaptable to generating real-time estimates during future epidemics. As we continue to build epidemiological capacity in developing nations, including South Africa, we must keep in mind the need for rapid assessments of transmissibility of novel pathogens, in addition to disease severity, to better inform public health interventions.
